# Structure and *in silico* simulations of a cold-active esterase reveals its prime cold-adaptation mechanism

**DOI:** 10.1098/rsob.210182

**Published:** 2021-12-01

**Authors:** Nehad Noby, Husam Sabah Auhim, Samuel Winter, Harley L. Worthy, Amira M. Embaby, Hesham Saeed, Ahmed Hussein, Christopher R. Pudney, Pierre J. Rizkallah, Stephen A. Wells, D. Dafydd Jones

**Affiliations:** ^1^ Department of Biotechnology, Institute of Graduate Studies and Research, Alexandria University, Alexandria, Egypt; ^2^ School of Biosciences, Molecular Biosciences Division, Cardiff University, Cardiff CF10 3AX, UK; ^3^ Department of Biology, College of Science, University of Baghdad, Baghdad, Iraq; ^4^ Department of Biology and Biochemistry, University of Bath, Bath BA2 7AY, UK; ^5^ School of Medicine, Cardiff University, Cardiff CF14 4XN, UK; ^6^ Department of Physics, University of Bath, Bath BA2 7AY

**Keywords:** serine esterase, molecular dynamics, enzyme structure, protein stability, structure–function

## Abstract

Here we determined the structure of a cold active family IV esterase (EstN7) cloned *from Bacillus cohnii* strain N1. EstN7 is a dimer with a classical α/β hydrolase fold. It has an acidic surface that is thought to play a role in cold-adaption by retaining solvation under changed water solvent entropy at lower temperatures. The conformation of the functionally important cap region is significantly different to EstN7's closest relatives, forming a bridge-like structure with reduced helical content providing greater access to the active site through more than one substrate access tunnel. However, dynamics do not appear to play a major role in cold adaption. Molecular dynamics at different temperatures, rigidity analysis, normal mode analysis and geometric simulations of motion confirm the flexibility of the cap region but suggest that the rest of the protein is largely rigid. Rigidity analysis indicates the distribution of hydrophobic tethers is appropriate to colder conditions, where the hydrophobic effect is weaker than in mesophilic conditions due to reduced water entropy. Thus, it is likely that increased substrate accessibility and tolerance to changes in water entropy are important for of EstN7's cold adaptation rather than changes in dynamics.

## Graphical abstract

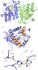

## 1. Introduction

Cold active/adapted enzymes are useful biotechnological tools due to their optimal activity at lower temperatures [1–4]. Moreover, their perceived thermolabile nature allows enzyme inactivation under mild temperature without altering product quality [5,6], such as texture and aroma in food industries. Esterases are particularly useful as they catalyse the hydrolysis of a wide variety of esters into their constituent alcohol and acid [7–11]. Cold-adapted esterases are important for temperature sensitive applications such as synthesizing fragile chiral compounds [12,13], cold washing laundry [14], environmental bioremediation [15] and the food industry [16].

There are several proposed molecular mechanisms by which enzymes adapt to working in cold conditions [11,17–20]. One is temperature-dependent dynamics, both globally across the whole enzyme and locally at regions important for function [19]. While dynamical flux is critical to enzyme function, cold active enzymes are thought to be inherently more dynamic, especially around the active site. This allows the continued structural flexibility at low temperatures so enabling substrate access, binding and turnover under low energy [10,21–23]. However, increased flexibility comes with an entropic cost leading to lower overall temperature stability resulting in the enzyme being more thermolabile. By contrast, the increased structural stability at higher temperatures of mesophilic and thermophilic enzymes can come at the expense of reduced activity at lower temperature through, for example, increased rigidity [21]. The features that contribute to altered dynamic properties include low levels of rigidifying residues (e.g. prolines), glycine clustering, low salt bridge and H-bond content, less densely packed hydrophobic core and few aromatic–aromatic interactions [10,19,24,25]. Additional mechanism has been proposed in which the protein is adapted to the change in water entropy [26]. As temperature drops, water molecules will become more organized and viscous so reducing the impact of the hydrophobic effect that is critical for maintaining a folded protein. Increased surface negative charge allows the protein to retain stable interactions and solvation with water under changed entropic and viscosity conditions [17,19]. In some cases, the oligomerization state of an enzyme affects its flexibility and activity under low temperature conditions [27,28].

Here we determine the structure of a novel hormone sensitive lipase (HSL) family IV cold active esterase (EstN7) isolated from *Bacillus cohnii* strain N1 [29] to understand the basis of cold adaptation. *B. cohnii* was originally isolated from leather industry effluents and is considered psychrotolerant. Unlike many esterases isolated from psychrophilic organisms that still have optimal enzyme activity at greater than 20°C, EstN7 is truly cold active with an optimal temperature of 5–10°C with a dramatic drop-off in activity above 20°C [29]. We have previously shown that EstN7 retained function in the presence of up to 30% of various organic solvents [29], which makes it potentially useful in a variety of applications, such as fine chemical synthesis and pharmaceutical industries. The natural function of the enzyme is currently unknown and little is known about its structure; the closest structural homologue is HerE, a putative heroin esterase from *Rhodococcus* sp. strain H1 [30]. The EstN7 structure revealed a protein with a highly acidic surface and an N-terminal cap region that formed a bridge-like structure allowing multiple channels for the substrate to access the active site. Analysis of EstN7's dynamics confirmed that the N-terminal cap was the most flexible region of enzyme in line with related esterases but the dynamic profile differed between the subunits. Overall, EstN7 was not appreciably more dynamic compared to its mesophilic and thermophilic relatives, which is counter to what might be expected for a cold-adapted enzyme.

## 2. Results

### 2.1. Structure of EstN7

**Figure 1.**
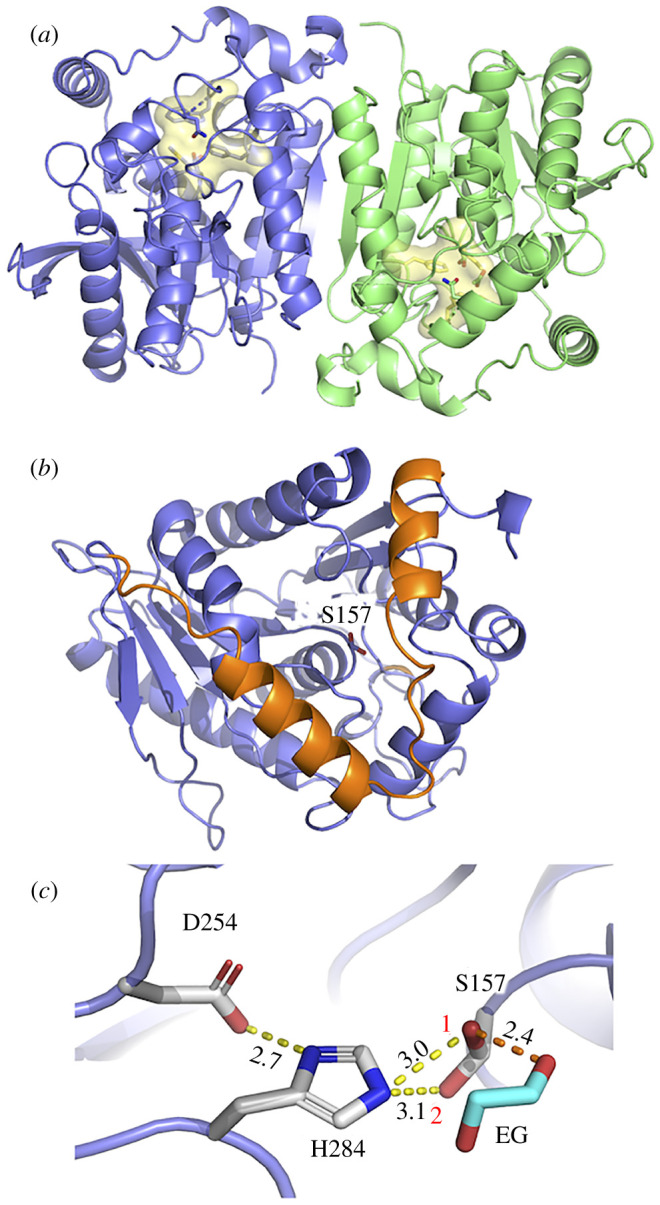
Structure of EstN7. (*a*) Overall structure of EstN7 with the A subunit in blue and B subunit in green. The active site is highlighted in yellow. (*b*) The A subunit with the N-terminal cap domain coloured orange and the nucleophilic S157 shown as sticks. (*c*) Catalytic triad with alternative rotamers of S157 labelled 1 and 2. An ethylene glycol (EG) is also shown as predicted to be present from electron density. Comparison with subunit B active site is shown in electronic supplementary material, figure S2*a*.

We determined crystal structure of EstN7 to 1.6 Å resolution (see electronic supplementary material, table S1 for statistics; PDB accession code 7b4q). EstN7 was observed as a dimer in the unit cell, which was confirmed in solution by size exclusion chromatography (electronic supplementary material, figure S1). EstN7 is a member of the HSL (or IV) family with a α/β hydrolase fold [31] and consists of a central eight stranded β-sheet surrounded by nine helices (figure 1*a*). The dimer interface area is 1049 Å^2^, equivalent to approximately 8% of the average monomer accessible solvent surface area (SASA). A total of 24 residues from each monomer contribute to the dimer interface, with 13 H-bonds, one salt bridge and a further 16 interactions classed as salt bridges/H-bonds according to PISA [32]. The two β-sheets are connected across the dimer interface via an antiparallel arrangement of strand 8 from each subunit to form effectively a single continuous sheet, with residues in the C-terminal helix and helix 8 also contributing. Each subunit is near identical with a 0.124 Å C_α_ root mean squared deviation (RMSD) and the catalytic residue occupying identical conformations (electronic supplementary material, figure S2).

The nearest structural homologues are dimeric mesophilic and thermophilic bacterial esterases: a putative heroin esterase (HerE) from *Rhodococcus* sp. strain H1 [30], a mycobacterial LipW [33] and the highly enantio-selective PestE from the thermophile *Pyrobaculum calidifontis* [34]; the sequence identities are 40%, 37% and 35%, respectively (see electronic supplementary material, figure S3a for sequence alignment). Structural alignments generated RMSD of 0.62 Å, 1.08 Å and 1.18 Å, respectively, confirming protein fold conservation (see electronic supplementary material, figure S3b for structural alignment). HerE and LipW represent mesophilic esterases while PestE is classed as a thermophilic esterase. An esterase from the marine bacterium *Thalassospira* sp. [35] is the closest putative psychrophilic structural homologue with a sequence identity of 28% and RMSD of 1.3 Å.

Within each individual EstN7 subunit, there are 29 salt bridges according to ESBRI server [36]. This is relatively high for a cold active enzyme, especially in relation to its mesophilic relatives (HerE, 21 salt bridges and LipW, 28 salt bridges). The distribution of non-covalent constraints identified by FLEXOME for rigidity analysis [37,38] is shown in electronic supplementary material, figure S4 and is consistent with the PISA and ESBRI analyses. Multiple non-covalent constraints are visible in the dimer interface that links the two subunits. Each subunit is rich in hydrophobic tether interactions, a total of 330 such interactions being found in the dimer. Polar interactions are also common in the dimer, including 54 with effective energies of −8 to −10 kcal mol^−1^; these are strong salt bridges.

**Figure 2.**
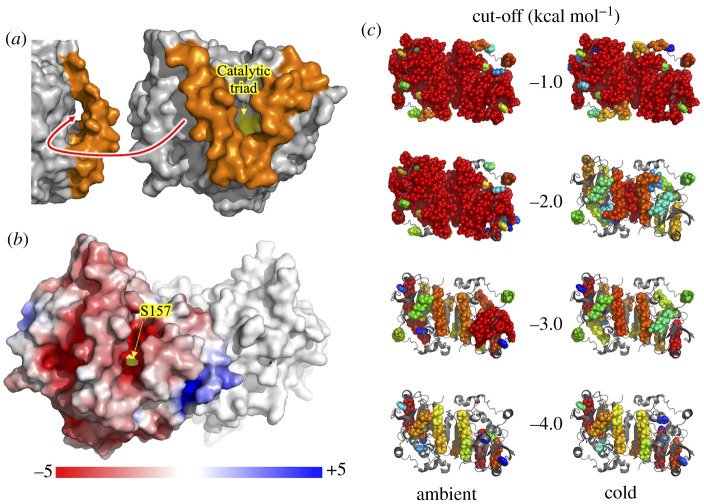
Surface and rigidity of EstN7. (*a*) Surface view of EstN7 with the cap region coloured orange and the catalytic triad at the bottom of a deep cleft coloured yellow. Shown left is an approximate 90° rotation to display a second channel. (*b*) Electrostatic potential surface of EstN7 subunit A calculated using APBS [39]. Colour scaling of electrostatic potential is shown. Rigidity features of EstN7 are shown in (*c*) with the 20 largest rigid clusters shown as spheres and coloured as rainbow from red (largest) to blue (20th largest). The ambient column shows results using a standard analysis at cut-offs from top to bottom of −1, −2, −3 and −4 kcal mol^−1^, while the cold column shows results when the weakening of hydrophobic tethers at low temperatures is taken into account. Flexible regions are shown as backbone cartoon and coloured grey.

As with other family IV esterases, EstN7 has an N-terminal cap structure. In EstN7 it comprises P7-D42 and covers the main *α*/β catalytic domain (figure 1*b*). The cap is generally the most dynamic component of the esterases and is thought to be important for substrate access and specificity as it forms the wall of channel leading to the active site [8,10]. Compared to related esterases, the second helical segment is shorter and further away from the first helical segment (electronic supplementary material, figure S5). For example, in EstN7 the second helical segment comprises 14 residues (V24-L37) whereas for HerE it is approximately one turn longer and comprised 18 residues (D25-A42), with PestE longer again comprising 21 residues (D25-N45). Analysis of EstN7 solvent accessible surface area (SASA) shows that the second helix-loop (residues 23–40) is not tightly associated with the catalytic domain as is the case in related structures but forms a bridge-like structure that reveals additional potential tunnels to the active site not present in the closely related mesophilic and thermophilic esterases (figure 2*a*). The bridge structure is anchored by M31 and L40 through hydrophobic interactions; the connecting residues do not form any additional interactions with the rest of the protein. In comparison, the same regions of HerE and PestE form extensive interactions with neighbouring residues including hydrophobic (e.g. I37, M40 and L41 in HerE and F33, L40 and V41 in PestE) and H-bonding (Y33 with S97 in HerE and E34 to S91 in PestE). Indeed, there is little sequence similarity in this region of the cap (electronic supplementary material, figure S3). The calculated volume of the active site using CASTp [40] is 1026 and 1148 Å^3^ for the A and B subunit, respectively. This is significantly larger than HerE (654 Å^3^), LipW (305 Å^3^) and PestE (637 Å^3^).

The active site lies at the bottom of the cleft formed by the cap region (figure 2*a*) and comprised Ser157, Asp254 and His284 (figure 1*c*). The nucleophilic Ser157 residue is in the conserved pentapeptide motif GXSXG between strands β5 and β6 common to family IV members. The EstN7 nucleophilic serine residue resides in the GQ**S**AG sequence motif, which puts it in the GDSAG sequence category rather than the less populated GTSAG class [41]. In both subunits, S157 adopts two distinct hydroxymethyl rotamers with approximately 115° between the two (figure 1*c* and electronic supplementary material, figure S2). Dual conformations of S157 have been observed previously for related esterases with the different rotamers thought to be representative of the catalytic cycle rather than an artefact of crystallization [30,33,42]; one rotamer represents the hydrolytic competent state while the other facilitates product release and prevents reverse reactions. Both rotamers are capable of polar interactions with His284 with distances of 3.0 Å and 3.1 Å. Rotamer 1 faces towards the acyl binding pocket as expected for its role in catalysis so is likely to represent the hydrolytically competent state. Electron density that equates to ethylene glycol was also observed in the active site of both subunits, with an additional ethylene glycol found in subunit B (electronic supplementary material, figure S2b). Rotamer 1 is closest to the observed ethylene glycol moiety bound in the active site within the alcohol binding pocket, with the hydroxyl group within H-bonding distance (2.4Å) of the alcohol group (figure 1*c*).

Ethylene glycol could thus act as a mimic for the alcohol product/substrate associated with esterase activity. The remaining residues comprising the catalytic triad, Asp254 and His 284, are found between β7-α8, and β8-α9, respectively. The oxyanion hole, which helps stabilize intermediates during catalysis, is located close by and is comprised of the conserved 83-HGGG-86 motif (electronic supplementary material, figure S2a).

Sequence analysis of EstN7 reveals a high proportion of charged residues, with acidic residues dominating (Asp–Glu:Lys–Arg ratio being 48 : 33). Surface electrostatics were calculated using APBS (Adaptive Poisson–Boltzmann Solver) [39] and revealed the surface of EstN7 is largely negative with a few basic patches (figure 2*b*). The substrate binding cavity is largely acidic in nature (figure 2*b*). The thermophilic PestE has a lower number of charged residues (68 versus 88 for EstN7) with a nearly 1 : 1 ratio of basic and acidic residues (Asp–Glu : Lys–Arg ratio being 35–33), which is manifested in its electrostatic surface profile (electronic supplementary material, figure S6).

### 2.2. Structural rigidity of EstN7

Rigidity dilution [43] provides information on the relative rigidity/flexibility of different parts of a protein crystal structure. In this process, polar constraints are gradually eliminated in order of strength by lowering a ‘cut-off’ value which excludes weaker bonds from the analysis. Flexible loop regions lose rigidity at small cut-offs whereas the ‘folding core’ of the protein retains rigidity longest. Previous studies on the rigidity of extremophiles [38,44,45] have been consistent with the ‘corresponding states’ hypothesis [46]; thermophilic and hyper-thermophilic enzymes are more rigid than their mesophilic counterparts at a given cut-off, and the rigidity of a thermophile at a large cut-off resembles that of a mesophile at a smaller cut-off. We therefore assessed the rigidity of EstN7 using pebble-game rigidity analyses at a series of cut-off values (−1, −2, −3 and −4.0 kcal mol^−1^). The resulting rigid cluster decompositions are shown in figure 2*c*.

On carrying out a conventional rigidity analysis under ambient conditions (the default for mesophilic proteins), the EstN7 structure appears very rigid (figure 2*c*, ‘ambient’ column). At cut-offs of −1.0 and −2.0 kcal mol^−1^, a single large rigid cluster extends across most of the body of the dimer, with only the cap regions appearing independently flexible. Even at a cut-off of −3.0 kcal mol^−1^ a large rigid cluster spanning a significant portion of chain B is visible in the rigid cluster decomposition. By −4.0 kcal mol^−1^ cut-off, only individual helices appear as large rigid clusters. This apparently enhanced rigidity in a cold-adapted enzyme could appear counterintuitive. However, a previous study including a citrate synthase from an Antarctic microorganism [44] shows how to resolve the discrepancy. In the conventional analysis, a hydrophobic tether removes two degrees of freedom from the molecule.

To reflect the weakening of the hydrophobic effect at lower temperature, a second analysis in which each hydrophobic tether removes only one degree of freedom (figure 2*c*, ‘cold’ column) gives more appropriate results for a cold-adapted enzyme. While a single large rigid cluster extends across most of the dimer at the smallest cut-off of −1.0 kcal mol^−1^, at −2.0 kcal mol^−1^ much of the structure is flexible, with individual rigid helices, and the remaining largest rigid cluster is found at the centre of the dimer, spanning the dimer interface. Both ‘ambient’ and ‘cold’ analyses concur that the cap regions are the most flexible parts of the EntN7 structure.

Comparative rigidity analysis with HerE, LipW and PestE is shown in electronic supplementary material, figure S7. For the thermophilic PestE, the monolithic rigidity observed at −1 kcal mol^−1^ cut-off is also observed at −2 kcal mol^−1^, which is indicative of greater structural rigidity normally considered important for stability at high temperatures. For mesophilic HerE and LipW, the rigidity profile at the −1 kcal mol^−1^ cut-off, with even weak polar interactions included as constraints, gives a monolithic rigid cluster that extends across the entire structure. At the −2 kcal mol^−1^ cut-off, there is still a large rigid cluster across the structure, but more regions of flexibility are now observed, including around the active site cleft (electronic supplementary material, figure S7). Thus, rigidity analysis indicates that the mesophilic homologues are less rigid than the thermophilic PestE, as expected. The rigidity of EstN7 appears comparable to that of PestE in an ‘ambient’ analysis, as both structures are rich in salt bridge interactions. However, when considering the ‘cold’ analysis of EstN7 (figure 2*c*), this indicates that the structure is less rigid than the mesophilic or thermophilic homologue.

### 2.3. EstN7 dynamics

**Figure 3.**
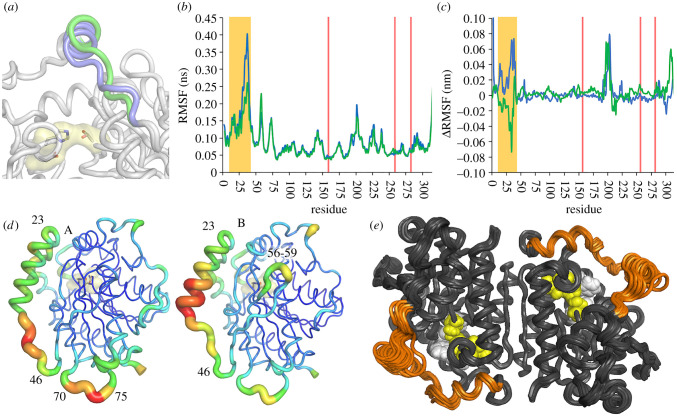
EstN7 dynamics. (*a*) Comparison of the conformation of the second helical component of the cap region in the A (blue) and B (green) subunit. The catalytic triad is shown as sticks for reference. (*b*) Residue-by-residue root mean squared fluctuation (RMSF) of C_^α^_ during 100 ns molecular dynamic simulations. Overlayed are the values for A (blue) and B (green) subunit. RMSF is reported as an average of five independent simulations run for 100 ns at 298 K (25°C). (*c*) RMSF difference between simulations run at 283 K and 308 K for the A and B subunit. The ΔRMSF was calculated by subtracting 283 K value from the 308 K value. A positive or negative value reflects increased RMSF either higher or lower temperature, respectively. The RMSF plots at each temperature are shown in electronic supplementary material, figure S8. The residues comprising the lid region are highlighted in the orange box and catalytic residues by the red line. Electronic supplementary material, movies 1–3 provide an additional representation of the backbone dynamics for a single run at 25°C, 10°C and 35°C, respectively. (*d*) Putty diagrams of the A (left) and B (right) subunits to represent the B-factor changes over the backbone. Higher values correspond with thicker, redder putty. B-factor values over the cap region are shown in figure 4. (*e*) An ensemble of 20 structures generated by geometric simulations of flexible motion biased parallel and antiparallel to the 10 lowest-frequency non-trivial normal modes, representing the amplitude of flexible motion in the structure achievable while maintaining its bonding and steric geometry. The catalytic triad is shown as yellow spheres and the plug residues as white, while the cap region is coloured orange. The greatest extent of conformational variation is seen in the cap regions, which can freely explore motions of several Ångström in amplitude.

Using a combination of B-factors and temperature-dependent molecular dynamics (MD), flexible regions of EstN7 were identified. In line with the rigidity analysis, both MD simulations and B-factors show clearly that the cap region is the most dynamic aspect of EstN7 (figure 3*a*–*c*), as is the case with esterases in general [7,10,31].

However, the cap region does appear to have different dynamics between the two subunits, including at different temperatures (figure 3*b,c* and electronic supplementary material, figure S8). While both subunits have the bridge-like structure associated with the second helical segment, the cap region is marginally closer to the catalytic domain in subunit A compared to B (figure 3*a*). In subunit A, the loop immediately following the second helix has the highest B-factors, whereas it is C-terminal half of the second helix for subunit B (figure 3*d*). In subunit A, a second region comprising residues 70–75 adjacent to the C-terminal end of the cap region display higher than average B-factors.

A similar trend was observed for MD simulations of EstN7 run at 298 K (figure 3*b*). The root mean squared fluctuations (RMSF) over the C_α_ atoms revealed that residues within the cap region are the most flexible regions in both subunits together with the adjacent 70–75 loop. The MD simulations suggests that the A subunit is more dynamic in the cap region around the second helical segment, which could imply this helical region has more scope for movement to potentially a more open conformation. MD also shows that the turn comprising residues 56–59 also has a higher dynamic profile, which is backed up by the increased B-factors in the equivalent region in subunit B (figure 3*b,c*). MD also suggests that a short region centred on residue 201, which is adjacent to the cap domain and close to the N-terminal (electronic supplementary material, figure S8c), is also flexible but B-factors indicate this region is not overly dynamic.

Simulations run at 283 K and 308 K (10°C and 35°C, respectively) show that much of the protein does not undergo any significant change in flexibility (figure 3*c* and electronic supplementary material, figure S8*a,b*). The short segment centred on residue 201 does appear to be more flexible at higher temperatures. The cap region does exhibit temperature depended changes in RMSF but as with the B-factors and 298 K MD simulation, the temperature-dependence of the cap region differs depending on the subunit (figure 3*c*). The cap region of the A subunit becomes more dynamic at the higher temperature whereas the B subunit is slightly less dynamic. Comparison of the RMSF data at each temperature (electronic supplementary material, figure S8*a,b*) suggests that at 283 K, the cap regions in each subunit have similar dynamics compared to the different regimes observed at the higher temperature.

**Figure 4.**
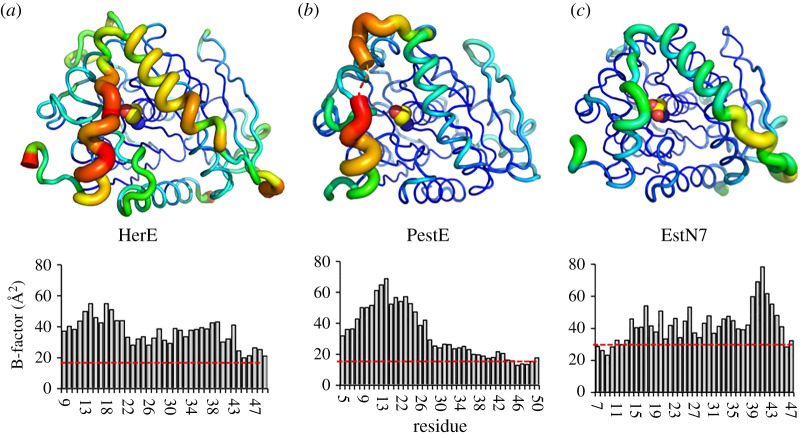
B-factor analysis of HerE and PestE. Relative changes in B-factor along the backbone for (*a*) HerE (PDB 1lzk) [30], (*b*) PestE (PDB 2yh2) [34] and (*c*) EstN7 (PDB 7b4q). Top panels are the putty diagram of the A subunit of each esterase. Putty diagrams were generated in PyMOL. The same scaling is applied across the three structures so represents a comparative analysis. The lower panel represents the Cα B-factor values for the cap region. The average Cα B-factor across the whole structure is shown as a red line (HerE, 18 Å^2^; PestE, 18 Å^2^; EstN7, 26 Å^2^). Residues 15–19 are missing from the PestE plot as they are absent in the PDB file.

We compared the general trend in C_α_ B-factors observed across the cap region for the A subunit of EstN7 with those found for the A subunits of HerE [30] and the thermophilic PestE [34] (figure 4). Overall B-factors for the cap regions for each of the esterases are higher than the average backbone value. EstN7 has an overall average B-factor approximately 26 Å^2^, which is higher than both PestE and HerE (both approx. 18 Å^2^). However, the change in the normalized B-factor for the cap region is lowest for EstN7 (1.6-fold) compared to PestE (1.9-fold) and HerE (2.0-fold). Thus, it appears that the cap domain of EstN7 is proportionately less flexible compared to even thermophilic relatives. In comparison with EstN7, the highest B-factor values for both HerE and PestE are in the first helical region of the cap structure (figure 4), with residues 15 to 19 missing from the structure of PestE presumably due to high mobility.

B-factors provide an experimental measure of local thermal fluctuations in a crystal structure, while MD simulations over nanosecond timescales probe the capacity for concerted local flexible motion. Geometric simulations of flexible motion [44,47,48] provide a complementary probe of the global, collective flexible motion achievable on longer timescales. In this approach, we first obtain low-frequency normal modes of motion for the protein, representing ‘easy’ directions of motion which the protein can explore with minimal restoring force or energetic penalty. We then explore motion of the structure along these directions while using constraints to retain the bonding geometry of the starting structure. We therefore carried out elastic network modelling of EstN7 on a one-node-per-residue basis with a spring interaction range of 10.0 Å, extracting the six trivial rigid body motions and the 10 lowest-frequency non-trivial normal modes (modes 07–16). Examination of the resulting eigenvectors showed that the two lowest-frequency non-trivial motions, modes 07 and 08, are collective twisting and bending motions of the entire dimer, while modes 09, 10 and higher are more localized on flexible loop regions and particularly in the cap regions. Geometric simulations biased parallel and antiparallel to each mode eigenvectors then generated an ensemble of flexible variations on the EstN7 structure, representing the amplitudes of motion achievable in these ‘easy’ directions of low-frequency motion while maintaining steric exclusion and the covalent and non-covalent bonding geometry of the crystal structure. This ensemble is shown in figure 3*e*. The capacity of the entire dimer for collective motion is very limited by the well-constrained dimer interface, with the central part of the dimer displaying little variation in the ensemble. By contrast, the cap regions clearly display a capacity for large-amplitude flexible motions which move the cap through amplitudes of several Ångström over the active site cleft below. Two low-frequency modes, 09 and 10, are particularly localized onto the cap regions; a visualization of these motions, and the consequent variations in the primary and secondary channel geometries, are shown in electronic supplementary material, figure S9.

MD simulations of HerE mirror the B-factor rigidity analysis (electronic supplementary material, figure S10). RMSF is similar between HerE and EstN7 over most of the backbone at both 10°C and 35°C suggesting little difference in the global dynamics between the two proteins. The average RMSF over the C_α_ atoms is 0.099 nm for HerE and 0.089 nm for EstN7 at 35°C, and 0.081 nm for HerE and 0.086 nm for EstN7 at 10°C. In the lid region, residues prior to and incorporating the first helix segment are more flexible in HerE at both temperatures compared to EstN7 while the second lid helix is more flexible in EstN7. Interestingly, MD simulations suggest that HerE is more flexible than EstN7 in the loop region immediately after the second helical region where there is a single amino acid insertion in HerE. Furthermore, HerE is more dynamic around residue 230–235 at both temperatures. This region also has higher B-factors values for HerE and corresponds to a three residue insertion in HerE compared to EstN7.

### 2.4. Thermal stability of EstN7

**Figure 5.**
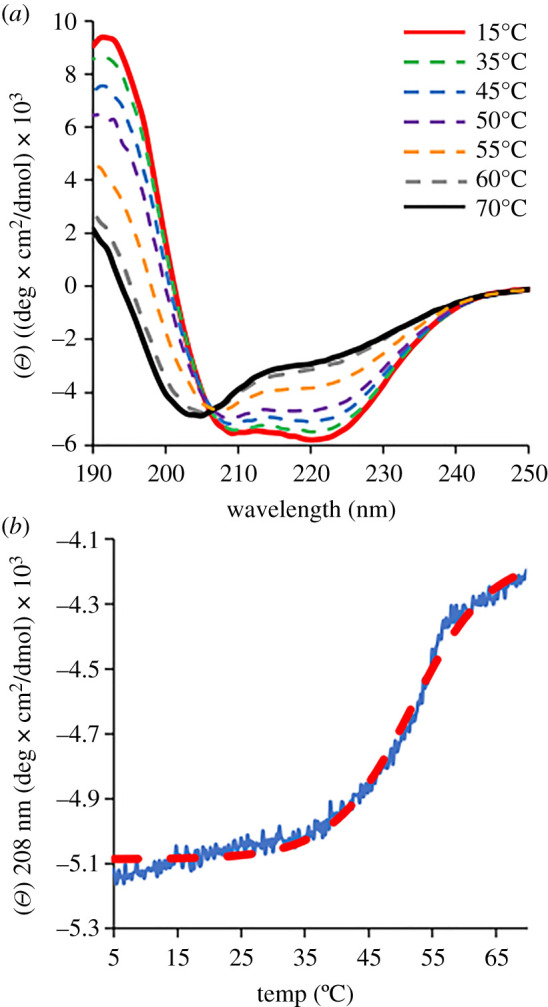
Thermal stability of EstN7 monitored by CD spectroscopy. (*a*) CD spectra of EstN7 at 15°C (red solid line), 35°C (green dashed), 45°C (blue dashed), 50°C (purple dashed), 55°C (orange dashed), 60°C (grey dashed) and 70°C (black solid line). (*b*) Temperature-dependent changes in molar ellipticity at 208 nm for EstN7. The red line is a Boltzmann sigmoidal curve fit to the major transition during temperature ramping.

Circular dichroism was used to assess the overall structural thermal stability of EstN7. At 15°C, EstN7 had a significant helical character with characteristic troughs at 210 and 222 nm suggesting the enzyme is largely folded; these troughs become shallower above 15°C but still suggest a largely fold enzyme up to 50°C (figure 5*a*). Between 50 and 60°C, EstN7 undergoes a major structural transition, with the enzyme largely unfolded at 60°C giving the CD spectra shown in figure 5*a*. Thermal unfolding of EstN7 monitored by CD at 208 nm revealed a major transition between 35 and 55°C, with a *Tm* of 51°C (figure 5*b*).

## 3. Discussion

While enzymes that work at low temperatures are important for a variety of processes, less is known about molecular basis for cold-adaption compared to thermophilic counterparts. Here, we determined the structure and dynamics of the cold active EstN7 [29]. Structural comparison with the closest mesophilic and thermophilic homologues has allowed us to propose mechanisms by which EstN7 functions optimally at low temperatures.

Many esterases are termed psychrophilic due to the organisms they derive from rather than their inherent cold-optimized activity, with the enzymes themselves commonly being mesophilic with regards to their optimal activity [10,35,49]. Previous work showed that EstN7 has optimum activity at 5–10°C, dropping off significantly between 20 and 30°C [29]. Thermal unfolding however suggests that EstN7 is relatively stable (figure 5; *Tm* = 51°C). It is generally thought that cold active enzymes tend to be thermally unstable, losing structure as well as function at relatively low temperatures primarily through increased dynamics and thus higher inherent entropy. However growing evidence suggests that this does not always hold true as shown here and elsewhere [17,23,28,50–52]. Thus, at least for these psychrophilic enzymes, relatively high global stability is not a physico-chemical restriction on cold activity. This in turn raises the question of the role of changes in global protein dynamic profiles as a common mechanism for cold adaptation, which is discussed below.

Protein dynamics are generally thought to play a critical role to protein adaptation to colder temperatures. While it is a commonly perceived that cold-adapted enzymes are inherently more flexible, it may not always hold true [53]; this appears to be the case here based on the global inherent dynamics of EstN7 and when compared to its mesophilic and thermophilic relatives. EstN7 does not have a particularly low level of rigidifying residues, such as prolines [54], with, for example, EstN7 having more proline residues (24) than the thermophilic PestE (20). Increased glycine content and clusters are also thought to increase flexibility; EstN7 and PestE have a comparable number of glycines (24 and 23, respectively), with the only glycine clusters associated with the conserved glycine-rich motifs of the oxyanion hole and nucleophilic serine. EstN7 also has a substantial number of salt bridges, with the mesophilic HerE having a lower number (29 versus 21, respectively). The hydrophobic cores of cold-adapted enzymes are also thought to be less well packed [19,24]. EstN7 has a well packed core rich in hydrophobic tether interactions. Indeed, EstN7 has a similar rigidity to the thermophilic PestE under ambient conditions, and more rigid than mesophilic relatives (electronic supplementary material, figure S7). MD analysis at 283 K and 308 K suggests that most of the residues do not significantly change in terms of their dynamic profile at the different temperatures (figure 3*c*).

The results of rigidity analysis and of geometric simulations of flexible motion are consistent with the above hypotheses. The rigidity analysis suggests that EstN7 at lower temperatures has rigidity comparable to mesophilic enzymes at ambient temperatures when the weakening of the hydrophobic interactions at low temperature is considered (figure 2*c* and electronic supplementary material, figure S7). During flexible motion along low-frequency normal mode directions the EstN7 cap regions are capable of substantial amplitudes of motion while retaining the local geometry of the crystal structure (figure 3*e*). This may in turn explain the counter-intuitive *T_m_* for EstN7 (figure 5). Temperature-dependent unfolding is based on increased entropy through increased conformational flexibility as temperature rises. The temperature at which entropy dominates enthalpy will result in a disordered unfolded protein is favoured over a folded protein; the more dynamic a protein is at low temperatures, the lower the temperature needed to unfold a protein. Globally, EstN7 is relatively rigid at low temperatures with a similar RMSF profile to its closest mesophilic relative (electronic supplementary material, figure S10), which may explain why a relatively high temperature needed for it to unfold.

In comparison to global dynamics across of the whole protein, changes in local dynamics, especially in regions important for catalysis, are also thought to be important for cold adaptation [17,21,23,55]. For example, while B-factors have been reported to be generally lower for cold-adapted proteins, these increase locally around regions important for catalysis [56]. A conformationally dynamic cap region is common to esterases and is important for activity [10]. In EstN7, the second helical component of the cap region is shorter than its closest thermophilic relative and has less helical content (figure 1*b* and electronic supplementary material, figure S5). In line with related esterases, B-factors (figure 3*d*), rigidity analysis (figure 2*c*), geometric simulations (figure 3*e*) and MD (figure 3*b,c*) show that EstN7 cap region is the most conformationally flexible part of EstN7, even at 10°C. While maintaining cap dynamics is likely to be critical to EstN7's low temperature activity, comparison of B-factors (figure 4) and MD simulations (electronic supplementary material, figure S10) does not suggest it is especially more dynamic compared to closely related esterases. Moreover, relative to the average B-factor over the whole protein, the cap region of EstN7 is comparatively less dynamic than two of its relatives. MD simulations show the C_α_ RSMF over the lid region is similar between EstN7 and HerE (0.163 nm for both proteins). Thus, it does not appear that dynamics of the functionally important cap region is significantly different to its closely related mesophilic and thermophilic structural relatives. MD simulations reveal that the dynamics of the cap region differs between the EstN7 subunits (figures 3*b,d* and electronic supplementary material, figure S8). At higher temperatures subunit A has an inherently more flexible cap region than B (figure 3*b*–*d*). Furthermore, relative change in dynamic profile of the cap region as a function of temperature is different in each subunit (figure 3*c*) and suggests that they coalesce to a similar profile in both subunits at 10°C (electronic supplementary material, figure S8). Thus, while a dynamic cap region is critical for EstN7 function, it does not need to be more flexible compared to its closest mesophilic and thermophilic homologues for optimal activity at lower temperatures.

The question thus arises, apart from dynamics, what is the basis for EstN7's optimal activity at low temperatures. Water entropy is key to protein thermodynamic stability, especially the hydrophobic effect, and its loss is important to the phenomenon of cold-denaturation. This is due to the change in water organization and viscosity at low temperatures and so there is a greater energetic cost to breaking the solvent H-bond network [26]. Protein surface charge is thought to play a role in addressing water entropy [19], with EstN7 having a high proportion of charged residues (25% of total residues are Arg, Asp, Glu or Lys), and an acidic surface in keeping with its high Asp/Glu : Lys/Arg ratio (figure 2*b*). Rigidity analysis of the EstN7 structure under ‘cold’ conditions (through weakening hydrophobic interactions to mimic the reduced hydrophobic effect due to lower water entropy) provides further evidence of water entropy as contributor to EstN7 cold adaptation (figure 2*b*). A negatively charged surface can offset water entropy by maintaining the critical solvation layer through favourable interactions with the water. Indeed, EstN7 is known to be tolerant to high concentrations of organic solvent [29]; the retention of a water shell may not only be advantageous for cold adaptation but may also be useful for solvent tolerance. Local high concentrations of charge could lead to charge repulsion and thus increased flexibility but both B-factors and MD simulations do not suggest this is the case here (figure 3); any charge repulsion can potentially be offset through interactions with water. It has been suggested that charged surfaces play a role in halo-tolerance [57] but EstN7 does not display any obvious salt tolerance (electronic supplementary material, figure S11).

**Figure 6.**
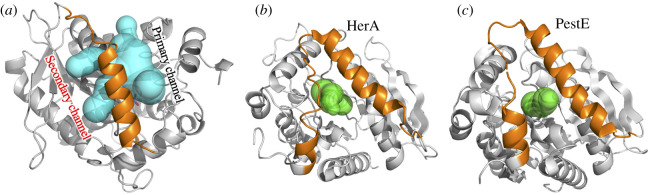
Available tunnels for access to the active site of (*a*) EstN7, (*b*) HerA and (*c*) PestE. Tunnels were calculated using CAVER 3.0 [58] with minimum probe radius set to 1.4 Å.

An additional mechanism for cold activity could be the conformation of the cap regions rather than solely its dynamics. Analysis of the protein's surface together with channel prediction suggests that the cap is partially detached from the main catalytic domain forming a bridge-like structure, with channels to the active site present from both sides (figures 3*a* and 6). These secondary tunnels are absent in related esterases (figure 6) despite the cap region following a similar path. The conformation of the cap domain in turn results in a wider main channel and the generation of additional tunnels to access the active site. By contrast, there is only one clear, common channel into the active site for the closely related structural homologues (figure 6*b,c*). As well as increased access routes, the active site cavity volume is greater than 1.7-fold larger for EstN7 than its closest mesophilic and thermophilic relatives. It has been proposed that increased cavity size is a mechanism for cold adaption and can promote substrate binding even at low temperature [28,59]. Overall, our findings are consistent with the hypothesis that the bridge-like structure of the cap region grants greater access to the active site than in related esterases.

## 4. Conclusion

Cold active esterases provide an insight into the molecular mechanism by which enzymes evolve to work in extreme conditions. EstN7 represents a novel cold-active esterase, with its nearest structural homologue having 40% or less sequence identity. A highly acidic surface profile helps retain structure when water becomes more viscous at lower temperatures. However, one perceived common feature of cold adapted enzymes does not appear to be critical here: higher local and global dynamics compared to mesophilic and thermophilic counterparts. Thus, EstN7's cold adaptation mechanism is likely to include dealing with change in water entropy and allowing improved access for substrate to the active site. Whether these combined features are common amongst esterase and enzymes in general remains to be seen but it does provide potential protein engineering routes to improve activity at lower temperatures for biotechnological applications and to probe further the molecular basis of cold adaptation.

## 5. Experimental procedures

### 5.1. Protein production and purification

Isolation of EstN7 encoding gene from *Bacillus cohnii* strain N1 and characterization of the recombinant enzyme were described previously [29]. EstN7 present within the pET28 a (+) plasmid was used to transform *Escherichia coli* BL21 DE3 cells. Single colonies were selected from LB agar plates supplemented with kanamycin and cultures left overnight in LB medium, which was used to inoculate 1 l fresh LB medium containing 35 µg ml^−1^ kanamycin. The culture was incubated at 37°C until reaching an A600 of 0.6 upon which EstN7 production was induced by the addition of isopropyl-β-thiogalactopyranoside (IPTG) to a final concentration of 1 mM; the culture was incubated for a further 18 h at 37°C. Cells were then harvested by centrifugation at 5000*g* for 20 min and the pellets washed and re-suspended in 50 mM Tris–HCl buffer, pH 8.0 (Buffer A). Cells were lysed by sonication for 10 min (eight cycles of 30 s, 18 000 Hz each with interval of 1 min on ice between cycles). Cell lysate was further clarified by through centrifugation at 10 000*g* for 10 min. Ni^2+^ affinity chromatography was used initially to purify recombinant EstN7, the cell lysate was separated from the cell debris. The crude mixture was loaded onto a nickel affinity chromatographic gel column (Sigma-Aldrich Co) and the column washed with buffer A containing 20 mM imidazole. EstN7 was eluted using buffer A supplemented with 500 mM imidazole. Samples were further purified using a Superdex 200 HiLoad 26/600 size exclusion column (Cytiva). The proteins were eluted using buffer A at room temperature. The resulting protein was highly pure, as judged by SDS PAGE (electronic supplementary material, figure S1*a*).

### 5.2. Structure determination

Crystallization conditions were explored using PACT premier™ and JSCG screens. The trials were performed in 96 well plates with a crystallization robot using sitting drop vapour diffusion method. For each trial, 1 µl of the protein sample (7 mg ml^−1^) and reservoir solution were mixed and equilibrated against 30 µl of the reservoir solution. The plates were incubated at 20°C and checked regularly for crystals formation. EstN7 crystals were obtained after 5 days in several crystallization conditions. The crystal producing the best diffraction was grown in 0.2 M lithium chloride, 0.1 M sodium acetate, 20% (w/v) PEG 6000, pH 5.0 (JCSG screen condition A05). Crystals were harvested by looping into thin nylon loops and plunged into liquid N2, for transfer to Diamond Light Source, Harwell, UK. Data were collected at beamline I03. Diffraction data were reduced with the XIA2 package using DIALS software. Scaling and merging were completed with AIMLESS and CTRUNCATE, part of the CCP4 package. The structure was solved with molecular replacement using PHASER [60], with HerE (1lzk.pdb [30]) as a starting model. Refinement with REFMAC [61,62] was alternated with graphics sessions in COOT [63] till convergence. Channels were identified using CAVER 3.0 [58] with a minimum probe radius of 1.4 Å and maximum distance of 10 Å.

### 5.3. Rigidity analysis, normal mode analysis and geometric simulations of flexible motion

Pebble-game rigidity analysis [37] (in which degrees of freedom are matched against constraints in a directed graph representing the covalent and non-covalent bonding network of the protein, identifying rigid clusters and flexible regions in the structure), elastic network normal mode analysis [64] (identifying directions of low-frequency flexible motion) and geometric simulations of flexible motion [47,48] (in which the structure is moved along a normal mode direction while maintaining its bonding geometry and steric exclusion) were carried out using a novel software implementation (FLEXOME) recently written by one of us (Wells). FLEXOME implements a body-bar version of pebble-game rigidity analysis which duplicates the functionality of the older FIRST software [37] and includes recently identified corrections to the handling of short salt bridge interactions [38]. The elastic network utility of FLEXOME provides the same functionality as standard codes such as Elnemo [64], but makes use of inverse iteration to extract only a limited number of the lowest-frequency modes rather than fully inverting the Hessian matrix. The geometric simulation utility of FLEXOME replicates the functionality of the FRODA [48] module, which was originally implemented within FIRST, and is written to explore flexible motion of the all-atom protein structure along low-frequency normal mode directions as has been previously done using the combination of FIRST/FRODA and Elnemo [44,47]. FLEXOME is available to researchers upon request from the repository at Bath (https://doi.org/10.15125/BATH-00940) subject to non-commercial license conditions.

In this instance, hydrophobic tethers and non-covalent polar interactions were identified by geometric criteria, and polar interactions were assigned effective energies from −0.0 to −10.0 kcal mol^−1^ based on the donor–hydrogen–acceptor geometry [38]. Rigidity analysis was then carried out using a progressively lowered cut-off to eliminate weaker hydrogen bonds, giving a ‘rigidity dilution’ [43] revealing the relative rigidity of different portions of the structure. In a standard analysis, polar interactions introduce five constraints ‘bars’ into the protein and hydrophobic tethers introduce two bars; each bar removes a degree of freedom from the system. In this case, we carried out both the standard analysis and a variant appropriate to cold conditions [44], in which hydrophobic tethers introduce only one ‘bar’.

The elastic network model was created by extracting one node per residue, using the C_α_ positions, and assigning springs of uniform strength between all pairs of nodes separated by a distance of less than 10 Å. Six rigid-unit motions of the structure were constructed analytically by FLEXOME and the 10 lowest-frequency non-trivial modes extracted by inverse iteration; these are denoted as modes 07–16.

Geometric simulations were carried out retaining the non-covalent constraint network selected by a cut-off value of −4.0 kcal mol^−1^, exploring motions parallel and antiparallel to each of modes 07–16. The bias in each step of the simulation was given by the (normalized) mode eigenvector multiplied by a scale factor of 0.1 Å; up to 500 steps were carried out in each simulation, with every 50th step saved in PDB format as a ‘frame’. The tolerance criterion on deviations from the bonding and steric geometry was 0.1 Å; each simulation halted after 500 steps or when the geometry could no longer be restored to within tolerances, which typically occurred within approximately 100–200 steps. The rigidity analysis, elastic network modelling and geometric simulation approaches are all rapid and computationally lightweight [47]; all of the above modelling was carried out on a laptop computer in a few CPU-hours.

### 5.4. Molecular dynamics simulations

MD on the dimeric EstN7 and HerE crystal structures were performed using Gromacs 4.6 [65] on the Hawk Supercomputing resource at Cardiff University (part of Supercomputing Wales). The pdb was initially converted to gmx format using the AMBER99SB force field [66]. The protein was then placed centrally in a cubic box at least 1 nm from the box edge. The protein system was then solvated with water molecules and total charge balanced to zero with Na^+^ and Cl^–^ ions. The protein was then energy minimized to below 1000 kJ mol^−1^ nm^−1^ with an energy step size of 0.01 over a maximum of 50 000 steps. The system was then temperature and pressure equilibrated before MD runs at 283 K, 298 K (for EstN7 only) or 308 K and 1 atmosphere pressure for 100 ns. Four independent MD simulations were chosen at each temperature for further analysis (see electronic supplementary material, figure S12 for RMSD over the course of the simulation for each EstN7 production MD run) with the C_α_ residue-by-residue root mean squared deviation fluctuation (RMSF) reported as an average of the four simulations.

### 5.5. Circular dichroism spectroscopy

Immediately prior to analysis, lyophilized EstN7 freeze dried in 50 mM potassium phosphate buffer, pH 7, was resuspended in water to 5 µM. Circular dichroism spectra were measured using 5 µM protein in approximately 5 mM potassium phosphate buffer, pH 7. Measurements were obtained at Bath University School of Chemistry on Chirascan CD Spectrophotometer (Photo Physics, Surrey, UK), using 0.1 mm pathlength quartz cuvette after blanking with 5 mM Tris pH 8.0. Absorbance was measured between 190 and 260 nm, at 1 nm intervals with a scan rate of 7.5 nm min^−1^. Full spectra were recorded at 5°C intervals starting at 15°C and ending at 70°C. The thermal melt (*Tm*) was determined by increasing temperature at a ramp rate of 1°C per minute using a Quantum Northwest Peltier and polarized absorbance at 208 nm was recorded at every 0.2°C change up to 70°C. Data obtained were converted to molar ellipticity and fitted to a Boltzmann sigmoidal curve in Origin V 2020 (MA, USA).

## Data accessibility

The structure of EstN7 has been deposited in the PDB under accession code 7b4q. FLEXOME is available to researchers upon request from the repository at Bath (https://doi.org/10.15125/BATH-00940) subject to non-commercial licence conditions. Other data associated with CD, SEC and MD are available from the repository at Cardiff (http://doi.org/10.17035/d.2021.0139101420).
